# Forensic analysis and evaluation of file‐wiping applications in Android OS

**DOI:** 10.1111/1556-4029.70174

**Published:** 2025-09-04

**Authors:** Dong Bin Oh, Somi Lim, Suji Lee, Yesong Jo, Gahyun Choi, Bumyun Kim, Huy Kang Kim

**Affiliations:** ^1^ School of Cybersecurity Korea University Seoul Korea; ^2^ Department of Computer and Information Engineering Kwangwoon University Seoul Korea; ^3^ Department of Information Security Engineering Soonchunhyang University Asan Korea; ^4^ Department of Computer Science and Engineering Seoul National University of Science and Technology Seoul Korea; ^5^ Department of Cyber Security Dankook University Yongin‐si Korea; ^6^ Information Communications Engineering Software and Communications Engineering Hongik University Sejong Korea

**Keywords:** Android forensic, anti‐forensics, application analysis, file‐wiping, forensic artifacts

## Abstract

Anti‐forensics refers to techniques designed to obstruct the discovery of evidence in digital forensic investigations. File‐wiping is one of the anti‐forensic techniques that make data recovery impossible by overwriting data with specific patterns. This technique poses significant challenges to investigators. Our study evaluates the effectiveness of file‐wiping applications on the Android OS from the anti‐forensic perspective. We selected six applications from the Google Play Store that support file‐level wiping. By analyzing these applications using reverse engineering and digital forensic tools, we addressed the three key research questions. First, we discovered that although one application claimed to provide file‐wiping functionality, it actually performed simple file deletions, making the deleted files recoverable. Second, we found that file‐wiping applications did not adhere to file‐wiping standards or guidelines. Lastly, by examining artifacts generated by the Android OS and applications during the file‐wiping process, we were able to reveal evidence of tool execution and artifacts of wiped files. Based on these findings, we propose a novel evaluation framework that assists digital forensic investigators in detecting traces of wiping activity and inferring information about deleted data on Android devices.

**Highlights**

- We analyzed six file‐wiping Android applications and suggested a novel evaluation framework.
- One wiping application performed file deletions, not wiping, which can recover the original file.
- Five file‐wiping applications did not follow published wiping standards and left forensic traces.
- We examined evidence of tool execution and artifacts of wiped files to track wiping activity.
- We identified novel forensic artifacts that enable evaluation of file‐wiping application activities.

## 1 INTRODUCTION

Anti‐forensics refers to techniques designed to prevent evidence from being discovered during digital forensic investigations. These techniques can be categorized into artifact wiping, data hiding, trail obfuscation, and attacks against forensics by their purpose technology [1]. File‐wiping is a technique that renders data unrecoverable by overwriting the data area with specific values, such as zeros or random data, unlike the deletion methods used in common filesystems. By applying file‐wiping, the subject of an investigation can avoid file recovery and spoil the digital evidence.

There are two main ways to make files hard to recover on mobile devices: factory reset or using a file‐wiping tool. While there has been extensive research on artifacts generated by file‐wiping in Windows [2] and factory reset in Android OS [3], there is a lack of research on file‐wiping applications on mobile devices. To respond effectively to file‐wiping, it is important to understand the artifacts left behind by file‐wiping tools. Also, the operation of wiping applications can vary; analyzing and categorizing traces from multiple wiping tools can help build prior knowledge. This would allow for more effective response even when encountering previously unknown wiping applications.

Investigation agencies need to determine whether file‐wiping has been performed on a mobile device and identify recoverable information. In civil litigation, the detection of data wiping is significant, as evidence of such actions may substantiate claims for damages based on spoliation.

### 1.1 Contribution

Our research makes several contributions to the field of digital forensics for law enforcement agencies, particularly in the context of Android OS anti‐forensic activities. According to statcounter [4], Android accounted for approximately 72% of the mobile OS market as of May 2025. This high market share is reflected in the volume of Android‐related cases in mobile forensics, highlighting the need for effective methodologies, particularly in anti‐forensics analysis that requires specialized expertise. With these findings from our research, we proposed a novel evaluation framework for file‐wiping applications that takes advantage of these artifacts to detect file‐wiping activities and infer information to identify the original data before data shredding effectively.

- Our experiments revealed that one application performed file deletions instead of file‐wiping, even though they asserted supporting file‐wiping, which leads to the recovery of the original file.
- We found that five file‐wiping applications did not follow the wiping algorithm standard. Although one application mistakenly overwrote data using random values rather than a single value due to developer error, the data still became unrecoverable.
- We identified and utilized previously unexplored forensic artifacts within the applications themselves, such as XML configurations and image caches, along with application execution artifacts that are already known in various studies.

In Section 2, we explain that anti‐forensics can be easily employed by installing applications with the necessary anti‐forensic techniques from the Google Play Store. Section 3 summarizes the research on wiping and anti‐forensics in Android. Section 4 describes the methodology and setup of the experiment for the study, as well as the research questions established to evaluate file‐wiping. Based on the research questions, Section 5 investigates whether the wiping function of the file‐wiping applications works effectively. Section 6 examines whether file‐wiping applications are implemented according to the guidelines for different algorithms. In Section 7, the study explores the artifacts generated during the execution of file‐wiping applications and how these artifacts can be used to detect file‐wiping. With these findings, we propose and test an evaluation framework in Section 8. Subsequently, Section 9 discusses the significance and limitations of this study, and Section 10 presents the results of the research.

## 2 ANTI‐FORENSIC TECHNIQUES FOR ANDROID DEVICES

### 2.1 Ecosystem of Android anti‐forensic techniques

Devices using the Android OS can download third‐party applications via Google Play Store. Through the Google Play Store, users can perform keyword searches and read the descriptions to choose the desired applications. iOS App Store applies stringent verification processes, making it difficult for applications that could harm the device to be listed [5].

From this perspective, anti‐forensic applications developed for Android can be easily downloaded and used.

### 2.2 Recoverable anti‐forensic techniques

Recoverable anti‐forensics refers to the possibility of recovering original data even after anti‐forensic techniques have been applied. In the Android operating system, recoverable anti‐forensic techniques include encryption and file‐hiding techniques. In the case of encryption, applications typically implement cryptographic algorithms with parameters such as an initialization vector (IV), a mode of operation (e.g., CBC or GCM), and a key size, which ensure the security and uniqueness of the ciphertext.

Most data encryption applications need to allow access to encrypted data even in states without external network connections, such as airplane mode, which means the decryption logic is embedded within the application itself [6, 7]. Through reverse engineering, the decryption logic can be reconstructed, allowing the recovery of the anti‐forensic encrypted data.

Similarly, file‐hiding applications use techniques such as encryption, changing file extensions, or moving files to hard‐to‐find locations to conceal data [8]. These techniques can also be countered by reverse engineering, which can uncover the concealed data through digital forensics [9].

### 2.3 Unrecoverable anti‐forensic techniques

Unrecoverable anti‐forensics makes it challenging to verify the original data even with digital forensics, as the original data are removed or altered. Timestomping [10] is a technique that interferes with the creation of a timeline during the digital forensic process by manipulating the timestamps of collected files.

In the Android environment, which commonly uses the EXT4 file system, timestomping can be achieved by modifying the *i_mtime, i_atime*, and *i_ctime* values within the inode structure. This can be done either through low‐level access to the file system (e.g., using a hex editor on a forensic image) or via higher‐level tools such as the *touch* command executed through Android Debug Bridge (ADB).

File‐wiping is a way in which the user overwrites the data area with specific patterns to make the data unrecoverable. File deletion in traditional filesystem only marks the file's metadata as deleted, allowing the storage area to be overwritten later. Even after partial overwriting with new data, remnants of the original data may remain recoverable. That is a phenomenon known as “file slack” [11]. However, file‐wiping ensures that the entire relevant area is overwritten for effectively preventing recovery [12].

## 3 RELATED WORKS

### 3.1 Data wiping in Android OS

In Blankesteijn et al. [3], end users often believe that a factory reset returns an electronic device to its original state, but forensic techniques can still recover user data in a post‐factory reset. This paper evaluates the efficacy of factory resets on Android devices running Android 11 or 12 by examining data before and after a factory reset and reveals that certain encrypted data remain accessible and partitions retain user‐generated data, which could expose user behavior.

Chaudhry et al. [13] explored the factory reset function, which is intended to delete all user data and restore the phone to its original state, complicating data recovery. However, the forensic acquisition of data remnants after a factory reset remains critical to investigators. Researchers find out the effects of factory resets on Android devices, and they detail the types of evidential artifacts that can still be found after factory resets.

Simon and Anderson [14] address the effectiveness of Android Factory Reset, analyzing 21 smartphones from 5 vendors across Android versions v2.3 to v4.3. They find that up to 500 million devices might not properly sanitize data partitions storing sensitive credentials, and up to 630 million may not adequately clear the internal SD card used for multimedia. Despite full disk encryption's potential to mitigate these risks, flawed resets may leave recoverable encryption keys.

Ahmad et al. [15] propose and develop a wiping application on Android devices, in accordance with the NIST SP 800‐88. To validate existing wiping applications, they installed three different tools on the Google Play Store with an Android version 8 device and tested 4 recovery tools. The experiment showed that the wiping applications did not operate according to standard algorithms, and data could be recovered.

Fatima et al. [16] examined the deletion performance of five image delete applications. They used forensic tools such as XRY, Autopsy, and DiskDigger to assess the extent of data recovery. The study also proposed the approach for locating completely deleted images within artifacts. Finally, they emphasized the importance of forensic tools in recovering images deleted by these applications.

Abozaid et al. [17] assessed the effectiveness of file‐wiping techniques in the Android environment by determining the extent of data deletion. Using the mobile forensic tool Belkasoft X and the anti‐forensic application AndroShredder, they found that 99% of the data were permanently deleted, supporting the claim that file‐wiping is the most effective technique to permanently delete data in mobile environments.

While Ahmad et al. [15] and Abozaid et al. [17] focused on the possibility of data recovery after using wiping applications, this study distinguishes itself by identifying the underlying causes of recovery and systematically analyzing implementation flaws in the applications as well as residual artifacts.

### 3.2 File‐wiping identification in Windows OS

Kim et al. [18] applied verification criteria to 23 file‐wiping tools and identified the top six tools that met all criteria. They found that prefetch files and registered registry keys generated during the installation, removal, and execution of each tool could be used to identify the specific file‐wiping tool and its manufacturer.

Oh et al. [2] researched to counter file‐wiping anti‐forensics using the NTFS transaction feature and machine learning. They used machine learning to predict which files had been wiped, which file‐wiping tools were used, and which file‐wiping algorithms were applied.

AlHarbi et al. [19] focused on analyzing the traces left by file‐wiping tools by examining how they alter the structure of metadata during file‐wiping. Using five file‐wiping tools, they demonstrated that the use of these tools on FAT32, exFAT, and NTFS filesystems could be identified.

Joo et al. [20] focused on the impact of file‐wiping tools on $MFT, $LogFile, and $DATA. Their study presented a comprehensive analysis of traces from 10 file‐wiping tools, proposing a method to detect file‐wiping traces by tracking various artifacts from the Windows OS.

Horsman [21] investigated the existence of “Digital Tool Marks” (DMT) by examining eight free deletion tools. They analyzed the impact of each tool on FAT32 and NTFS at the filesystem level, explicitly mentioning the deletion algorithms supported by each tool.

Lee et al. [22] researched to detect user behaviors related to data deletion tools and algorithms on the Windows Resilient FileSystem (ReFS) 3.7. They selected the 12 data deletion tools and extracted the deletion patterns supported by each tool. They developed a tool to identify deletion tools based on the extracted patterns.

### 3.3 Wiping algorithms

Pecherle et al. [23] proposed an algorithm to protect sensitive personal information by monitoring hard disk drives and applying file‐wiping algorithms instead of Windows OS standard deletion functions to files identified as containing sensitive information through keyword searches.

Wang and Zhao [24] categorized data deletion into five types and summarized the functions and deletion algorithms of popular tools. They introduced Quick Erase, a new data deletion algorithm that surpasses the speed of existing international data deletion algorithms and can be easily combined with various standard algorithms to create a variety of high‐speed mixed deletion algorithms.

Hasa et al. [25] reviewed the data deletion implementation of DoD 5220.22‐M and UK HMG IS5 E. They compared these implementations using performance tests, forensic tests, and data recovery tests. The performance test results indicated that the algorithms are significantly influenced by the anti‐forensic tools used. The recovery test results claimed that the UK HMG IS5 E, when used with the Active KillDisk tool, was the most secure deletion.

Various data sanitization standards define procedures for securely erasing digital data to prevent recovery. These standards specify the number of overwrites, the patterns to be used, and verification steps. Table 1 summarizes widely recognized wiping standards.

**TABLE 1:** Well‐known data‐wiping algorithms.

Standard name	Number of passes	Overwrite patterns
NIST SP 800–88 Rev.1	1+	Fixed value (e.g., 0x00)
DoD 5220.22‐M (E)	3	Fixed, Complement, Random
DoD 5220.22‐M (ECE)	7	Fixed, Complement, Random, Fixed, Fixed, Complement, Random
Gutmann method	35	Mixed various patterns
Schneier method	7	0x00, 0xFF, Random, Random, Random, Random, Random
UK HMG IS5 (Enhanced)	3	Zero, Random, Random
German VSITR	7	Zero, One, Zero, One, Zero, One, 0xAA

## 4 METHODOLOGY AND EXPERIMENTS

### 4.1 Methodology overview

In our study, we collected file‐wiping applications in the Google Play Store. To systematically evaluate the effectiveness of the file‐wiping functionality provided by these applications, we set up the following research questions:

- RQ 1: Do file‐wiping applications really render files completely unrecoverable?
- RQ 2: Do the file‐wiping algorithms implemented in the file‐wiping applications comply with the guidelines?
- RQ 3: Can traces of files wiped by the file‐wiping applications be discovered?

To address these questions, we employed a combination of static analysis, dynamic analysis, and artifact analysis. The detailed procedures are described in the following section.

### 4.2 Experimental procedure

We first conducted static analysis by reverse engineering on each file‐wiping application to obtain its decompiled source code. We used *dex2jar* to decompile the file‐wiping application and examine “wiping function.” This enabled us to inspect the implemented wiping algorithms, verify whether actual overwriting occurs, and identify any code paths that may leave forensic traces.

Based on this analysis, we formulated hypotheses addressing the three research questions: **(RQ1)** whether file‐wiping is functionally performed; **(RQ2)** whether the wiping algorithms conform to well‐known standards; and **(RQ3)** whether digital forensic artifacts are left behind.

To validate these hypotheses, we used *Frida*, a dynamic instrumentation toolkit widely used in reverse engineering. Frida allows runtime hooking of functions and variables within Android applications without modifying the APK itself [7]. This technique enabled us to observe actual application behavior during file‐wiping operations.

For RQ1, we compared memory content at known offsets before and after wiping to check data recoverability. For RQ2, we intercepted function calls related to data overwriting to confirm the number of passes and patterns used. For RQ3, we examined three types of artifacts: (1) execution traces at the OS level, (2) internal app‐generated metadata such as SharedPreferences, and (3) cached image files that may reveal contents of wiped files.

These combined analyses allowed us to assess the actual behavior and effectiveness of each file‐wiping application, beyond their claimed functionalities.

### 4.3 Experimental environment

We searched the Google Play Store using the terms “data wiping”, “complete delete”, and “file shred” resulting in 67 candidate applications. Most of these applications were general‐purpose cleaners or uninstallers, which did not support file‐level wiping and thus were excluded from this study. If an application met the criteria but required payment (e.g., Pro version), we bought it to include it in the experiment. From these, we selected six that met the following criteria:

1. file‐wiping functionality clearly indicated in the app's description (exclude 37 apps)
2. implementation of at least one wiping algorithm (exclude 16 apps)
3. over 5000 downloads (exclude 8 apps)

Although wiping at the partition level is also effective for data removal, it risks deleting unintended files just like factory reset [3], making the use of third‐party applications unnecessary [26]. Thus, we focused on apps that primarily support file‐level wiping, even if they also offer partition wiping as an optional feature. The final list of selected applications is shown in Table 2.

**TABLE 2:** List of application collected file‐wiping applications in Google Play.

Name	Version	Num of download	Last update	Num of reviews	Star ratings	Num of wiping algorithm
iShredder Standard	7.0.12	Over 1 million	2024. 2. 2.	Over 7000	4.6	25
Shreddit—Data Eraser	4.7.24109	Over 1 million	2024. 1. 14.	Over 7000	4.5	9
Data Eraser App—Wipe Data	1.4	Over 100 K	2024. 6. 3.	Over 2000	4.0	25
SDelete—File Shredder (Pro Version)	9.1	Over 100 K	2023. 5. 23.	Unknown	Unknown	5
Secure Wipe Out—Data Shredder	1.2.1	Over 10 K	2022. 3. 29.	Unknown	Unknown	5
ZERDAVA File Shredder	1.01.7725	Over 5 K	2021. 5. 28.	Unknown	Unknown	22

The experiments were conducted on three Android devices: Samsung Galaxy Note 9 (SM‐N960N, Android 9, Build: N960NKSU3CSJ1), Google Pixel 5 (Android 13, Build: TQ2A.230305.008.C1), and Huawei Redmi Note 7 (Android 10, Build: QKQ1.190910.002). All devices were rooted to allow access to system directories and observation of application behaviors.

To ensure generalizability and reduce device‐specific bias, we focused on artifacts that were observed across all three devices, excluding those generated by pre‐installed manufacturer‐specific apps (e.g., *Samsung Gallery album*).

We also limited our analysis to the logical level due to the presence of File‐Based Encryption (FBE) and Full Disk Encryption (FDE), which render physical‐level imaging (e.g., NAND or eMMC) less useful in modern Android devices.

All experiments were conducted in an environment consisting of Mac OS 14.4.1, Python 3.9, and Frida 16.1.4.

## 5 CLAIMED VS. ACTUAL: EFFECTIVENESS AND RELIABILITY ANALYSIS OF FILE‐WIPING APPLICATIONS

### 5.1 Static analysis

**FIGURE 1:**
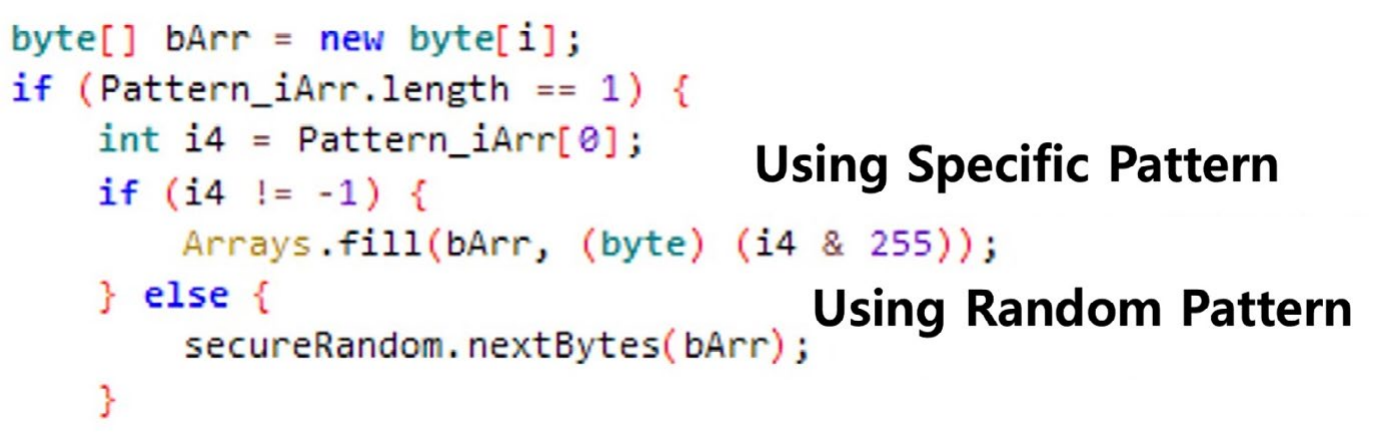
Figure shows typical file‐wiping function has overwrite function. This function uses two different kinds of pattern to overwrite data: Random and specific value. Through access file location and overwrite meaningless data, it is hard for the forensic investigator to recover original files.

To ensure that files cannot be recovered by digital forensic tools, file‐wiping must involve overwriting the data area of the file with meaningless values, rather than simply marking that the status of the file is “deleted” using metadata. The source code of six applications indicated that they performed file‐wiping by overwriting data areas with specific patterns, as illustrated in Figure 1.

**FIGURE 2:**
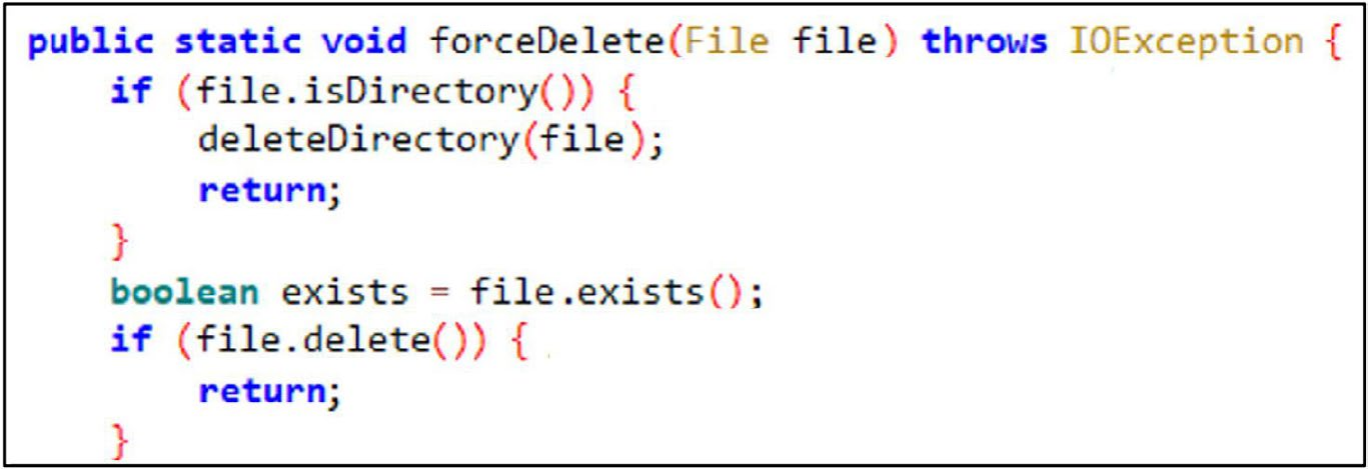
Figure shows the existence of a file delete function in secure wipe out, the file‐wiping application. The file‐wiping application itself does not need to include such a function; however, this file‐wiping application did not use the implemented file‐wiping function and instead used the file delete function.

**FIGURE 3:**
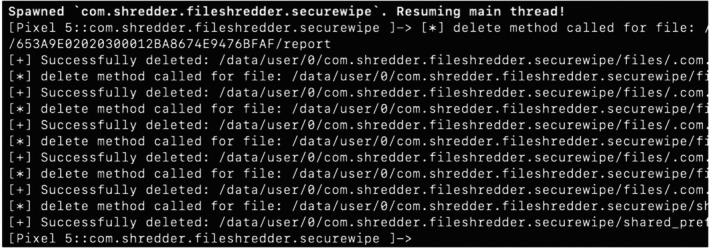
To verify the action of the forceDelete function, we used Frida to trace the behavior of Secure wipe out. We figured out there is no way to execute the implemented file‐wiping function and only the file delete function.

However, in the case of **Secure Wipe Out—Data Shredder** (hereafter referred to as Secure Wipe Out), it internally utilized the delete() method in its *deleteDirectory()* function, which only removed file metadata without overwriting actual data, as depicted in Figure 2. To verify this, we used Frida to hook the *forceDelete* function of Secure Wipe Out. We revealed that the application was executing a file deletion instead of using overwriting file contents. This is illustrated in Figure 3.

In the case of the **Data Eraser App—Wipe Data** (hereafter referred to as *Data Eraser App*), we found that it uses the RandomAccessFile class to access the actual data of files. The wiping pattern is a byte array filled with random values generated by the SecureRandom class.

**ZERDAVA File shredder** (hereafter referred to as *ZERDAVA*) used the getContentResolver() method within the fileOutputStream class to gain access to the file's data area. To consist of the random value pattern, the ZERDAVA utilizes the Random class.

**iShredder standard** (hereafter referred to as *iShredder*) obtains a channel to the data area of the file to be deleted using the fileOutputStream class. Then, iShredder overwrites the data with a random byte array generated using the SecureRandom class.

**Shreddit—Data Eraser** (hereafter referred to as *Shreddit*) accesses the file using the FileOutputStream class and overwrites it with a predefined pattern, generating random values using Java's Random class.

**SDelete—File Shredder** (Pro Version is used, hereafter referred to as *SDelete*) uses two ways to access the file with the getFilePointer() method of the RandomAccessFile class and obtain the FileDescriptor through the fileOutputStream class. To build the random pattern, SDelete uses the SecureRandom class to declare the byte array.

In general, a proper file‐wiping application must directly access the data area of the file to be wiped. However, if only the delete() method of the File class is used, it is impossible to directly access the data area, thus failing to achieve the intended purpose of file‐wiping.

### 5.2 Dynamic analysis and artifact analysis

We found that “Secure Wipe Out” implemented file deletion using the delete() method of the Java File class. To explore the difference between using the delete() method and file‐wiping algorithms, we conducted an experiment. We placed sample JPEG files on the device, recorded their offsets with binary data, and then executed all file‐wiping applications. We checked the data at the recorded offsets that had been changed.

**FIGURE 4:**
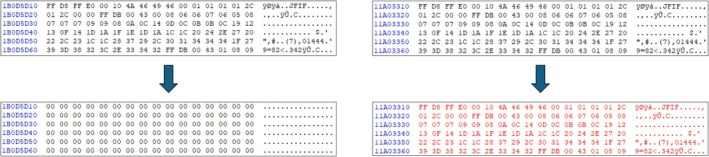
This figure shows the possibility of recovering deleted files in real‐world Android phone when using the file delete function in JAVA. Instead, in the case of using the file‐wiping function, the whole file data have been overwritten, so it is impossible to recover the original file.

In Figure 4, the left‐side figure demonstrates the result of complete deletion using “0x00” pattern wiping, while the right‐side figure shows the result of using the same feature in Secure Wipe Out. The comparison reveals that, although Secure Wipe Out was set to use the wiping function, it marked the file as deleted in the filesystem metadata without performing true wiping. Consequently, the original data remained intact. This residual data can be recovered through file recovery or file carving.

**FIGURE 5:**
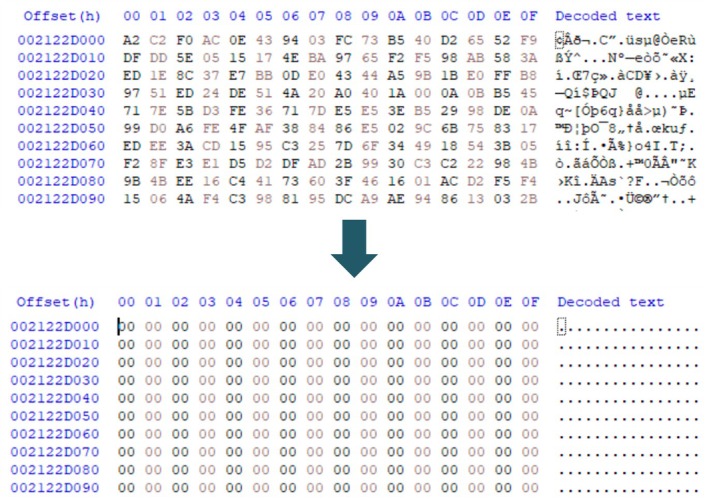
This figure shows how the file‐wiping function can affect File‐Based Encryption (FBE) Environment, which have been used in modern Android OS. The result of file‐wiping in the File‐Based Encryption Android environment also overwrites with patterns as “0.”

Results on devices employing File‐Based Encryption (FBE) were similar to those obtained from devices without FBE. In Figure 4, the JPEG file header is visible. However, in Figure 5, where FBE is applied, it shows no identifiable file structure; only the encrypted content is visible. After wiping, the predefined pattern overwrites the encrypted file content. These results demonstrate that while proper file‐wiping applications effectively overwrite data, applications like Secure Wipe Out, which merely delete files without overwriting, leave the original data susceptible to recovery.

A comparative summary of the file‐wiping methods implemented by each application is presented in Table 3.

**TABLE 3:** Comparison of wiping function in file‐wiping applications.

Application	Access file data	Randomness source	Actual wiping
Secure Wipe Out	File.delete()	None	Not implemented
Data Eraser App	RandomAccessFile.seek()	SecureRandom	Implemented properly
ZERDAVA	getContentResolver().openOutputStream()	Random	Implemented properly
iShredder	FileOutputStream.write()	SecureRandom	Implemented properly
Shreddit	FileOutputStream.write()	Random	Implemented properly
SDelete	RandomAccessFile.getFilePointer()	SecureRandom	Implemented properly

## 6 STANDARDS COMPLIANCE: VERIFYING FILE‐WIPING IMPLEMENTATIONS AGAINST GUIDELINES

### 6.1 Static analysis

In this section, we evaluate how well the wiping algorithms comply with current guidelines. We have analyzed the wiping algorithms within the applications to determine if each algorithm was correctly embodied according to the provided guidelines.

For Secure Wipe Out, as shown in Figure 1, it includes codes that overwrite the data area with specific numbers or random patterns; our previous experiments have revealed that it has only a file delete operation. We conclude that it does not comply with the guidelines; thus, we exclude it from further analysis.

In the Data Eraser App, the supported algorithms are defined in “com.protectstar.shredder.shred.methods.algorithms,” where each overwrite cycle is associated with a specific pattern. Among the 25 supported wiping algorithms, 23 algorithms are publicly documented and associated with established standards or guidelines. Of these, 14 algorithms are implemented in accordance with the prescribed specifications. Despite not using the exact patterns required by the guidelines, the number of overwrite cycles is correctly programmed in the Data Eraser App.

**FIGURE 6:**
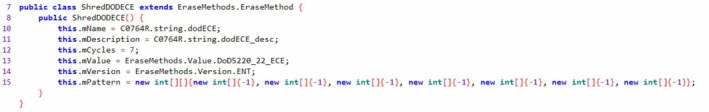
This figure shows violation in US DoD 5220.22‐M wiping guideline in Data Eraser App, the file‐wiping application. Its pattern shows all of the cycle overwrite with random represented as “−1.”

For example, Figure 6 illustrates the US DoD 5220.22‐M (ECE) [27] used in the Data Eraser app. However, the implementation only uses a “−1” as a random pattern to overwrite seven times, despite claiming to use the US DoD 5220.22‐M (ECE) (see Table 1).

In ZERDAVA, the wiping algorithms are implemented through custom functions rather than external libraries. Among the 22 supported algorithms, 19 algorithms comply with established standards, while two are undocumented and one does not follow the guidelines. This indicates that most of ZERDAVA's wiping algorithms accurately adhere to recognized standards.

In Shreddit, wiping algorithms are implemented through internal functions. Of the nine supported algorithms, all are based on publicly known standards or guidelines. Seven of them fully comply with their respective specifications, while two—VSITR (German standard) and NIST SP 800‐88 [28]—deviate from the standards in terms of overwrite cycles and pattern requirements.

SDelete supports five wiping standards. Except for one standard, all were developed according to the recommended standards.

**FIGURE 7:**
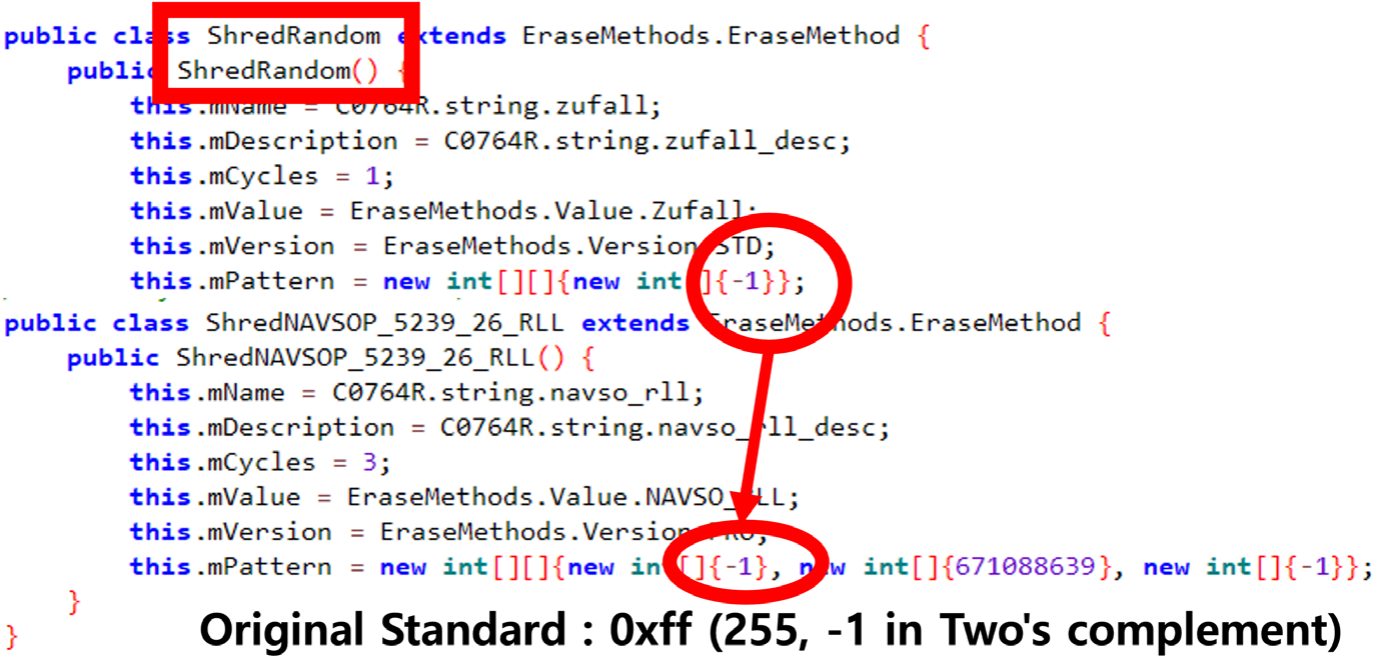
This figure shows the developer's mistake in iShredder, which represents −1 (random wiping) as 0xFF (Value wiping). It goes to overwrite the area with “0xFF,” not the random pattern.

iShredder uses the same library “com.protectstar.shredder.shred.methods.algorithms” as the Data Eraser App. Both applications contain an implementation issue where the value 0xFF is mistakenly represented as −1 due to two's complement encoding. Although the function was previously used “−1” for generating random patterns, this results in a deviation from the standard, as shown in Figure 7. Correcting the implementation to use 0xFF as intended would bring it into compliance with the relevant wiping standard.

### 6.2 Dynamic analysis and artifact analysis

**FIGURE 8:**
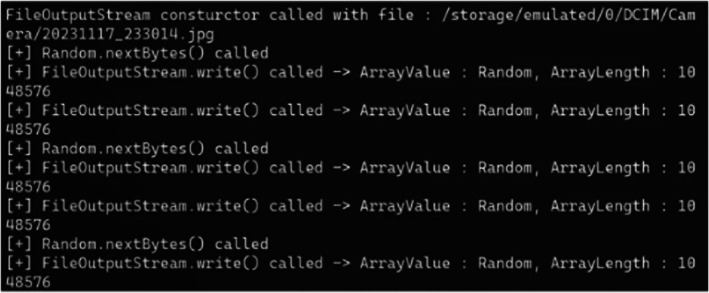
With Frida, we analyzed all of the parameters within the wiping function in each file‐wiping application. The figure is an example of the data gathering process.

Based on the findings of the static analysis, we conducted further analysis using Frida's function hooking shown as Figure 8. We hooked functions related to generating random values, such as Random and SecureRandom, and functions involved in assigning specific variables and overwriting the original data area. By monitoring whether these hooked functions were called and examining the parameter values used, we verified that the wiping algorithms operated as described in the static analysis.

While the final overwritten state can be observed in artifacts, this dynamic analysis was crucial for verifying each step of the wiping process. Through function hooking, we could verify whether applications actually performed multiple overwrite passes as required by guidelines such as DoD 5220.22‐M.

On average, we found that approximately 50% of the wiping algorithms implemented in each application complied with the relevant guidelines. Even when guidelines are not strictly followed, the difference between guidelines and implementations is small. There is a difference in the number of cycles or the patterns used. These deviations still ensure that all of the file data are overwritten with specific patterns at the same location and size as the original data, leaving no part of the original file data. Thus, the file‐wiping application meets the anti‐forensic purpose to make the data unrecoverable.

Table 4 summarizes the experimental results obtained by comparing the functions implemented in the applications with representative wiping standards such as single‐pass patterns, the US DOD 5220.22‐M (ECE), and the standard proposed by Bruce Schneier [29] and Peter Gutmann [30].

**TABLE 4:** Compliance result on wiping algorithm.

Name	Num of wiping algorithm	Num of unknown guidelines/standards	Num of algorithm comply with guidelines/standards	Comply with 1 pass wiping	Comply with US DOD 5220.M (ECE)	Comply with Bruce Schneier	Comply with Peter Gutmann
iShredder	25	2	14	O	X	X	X
Shreddit	9	0	7	O	O	O	Not support
Data Eraser App—Wipe Data	25	2	14	O	X	X	X
SDelete	5	0	4	O	Not support	Not support	Not support
ZERDAVA File Shredder	22	2	17	O	O	X	O

## 7 DIGITAL FORENSIC ARTIFACTS: DETECTING AND ANALYZING TRACES OF FILE‐WIPING ACTIVITIES IN ANDROID OS

### 7.1 Static analysis

This section focuses on the traces that can be utilized in digital forensics after a file‐wiping application has been executed. Therefore, instead of static analysis, which can be performed without running the application, we conducted dynamic and artifact analysis.

### 7.2 Dynamic analysis and artifact analysis

Application execution artifact. Even if a file‐wiping application is installed, it can be removed without being executed. However, if there is no installation, it cannot run. Therefore, to detect file‐wiping activities, we focus our research on artifacts that indicate the execution of applications. On Android, the primary artifacts that can prove the execution of an application are UsageStats, Recent Tasks, and Snapshots.

UsageStats is an Android system component that automatically tracks application usage. The default path for UsageStats is “/data/system/0/usagestats/” [31]. Within this directory, there are subdirectories named yearly, monthly, weekly, and daily, each containing files that include application usage statistics and timestamps for various activities. Using Android Debug Bridge (ADB), you can retrieve this information by running the command *adb shell dumpsys usagestats* or by parsing the protocol buffer structure at the storage path [32]. This allows you to obtain the package name of the file‐wiping app, the most recent execution time, total execution time, the number of times the app was started, and recent events associated with the application.

**FIGURE 9:**
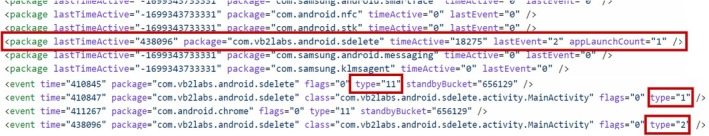
With usagestats, we can find out the execution time and name of the file‐wiping application, like this figure, which is related to the application execution artifact.

For example, considering “/data/system/0/usagestats/169934373331” with the content shown in Figure 9, we can identify the package name of SDelete as “com.vb2labs.Android.sdelete.” We figure out the filename as a baseline UNIX timestamp and add the value of event time to calculate when the event occurred. We can interpret UsagesStats as described in Table 5.

**TABLE 5:** Interpretation of UsageStats artifact in Figure 9.

Event time (GMT +0)	Event type	Class
438096 (2023/11/07, 08:02:51.427)	LastTimeActive	–
410845 (2023/11/07, 08:02:24.176)	11 (STANDBY BUCKET CHANGED)	–
410847 (2023/11/07, 08:02:24.178)	1 (MOVE TO BACKGROUND)	MainActivity
438096 (2023/11/07, 08:02:51.427)	2 (MOVE TO FOREGROUND)	MainActivity

In the case of Recent Tasks, the artifacts allow users to view the history of recently executed applications. They are stored in “/data/system_ce/0/recent_tasks” [33]. **Recent Tasks** enable users to easily switch between applications and continue from where they left off. From a digital forensic perspective, Recent Tasks can provide the names of the recently used packages and their last usage time, which can help us identify traces of file‐wiping application usage.

Snapshots, stored in “/data/system_ce/0/snapshots,” save JPEG images of the last screens displayed by recently used applications. Each file is named based on the task ID assigned to each process in Recent Tasks. This feature allows forensic investigators to view the last screen displayed on the usage of the application.

**FIGURE 10:**
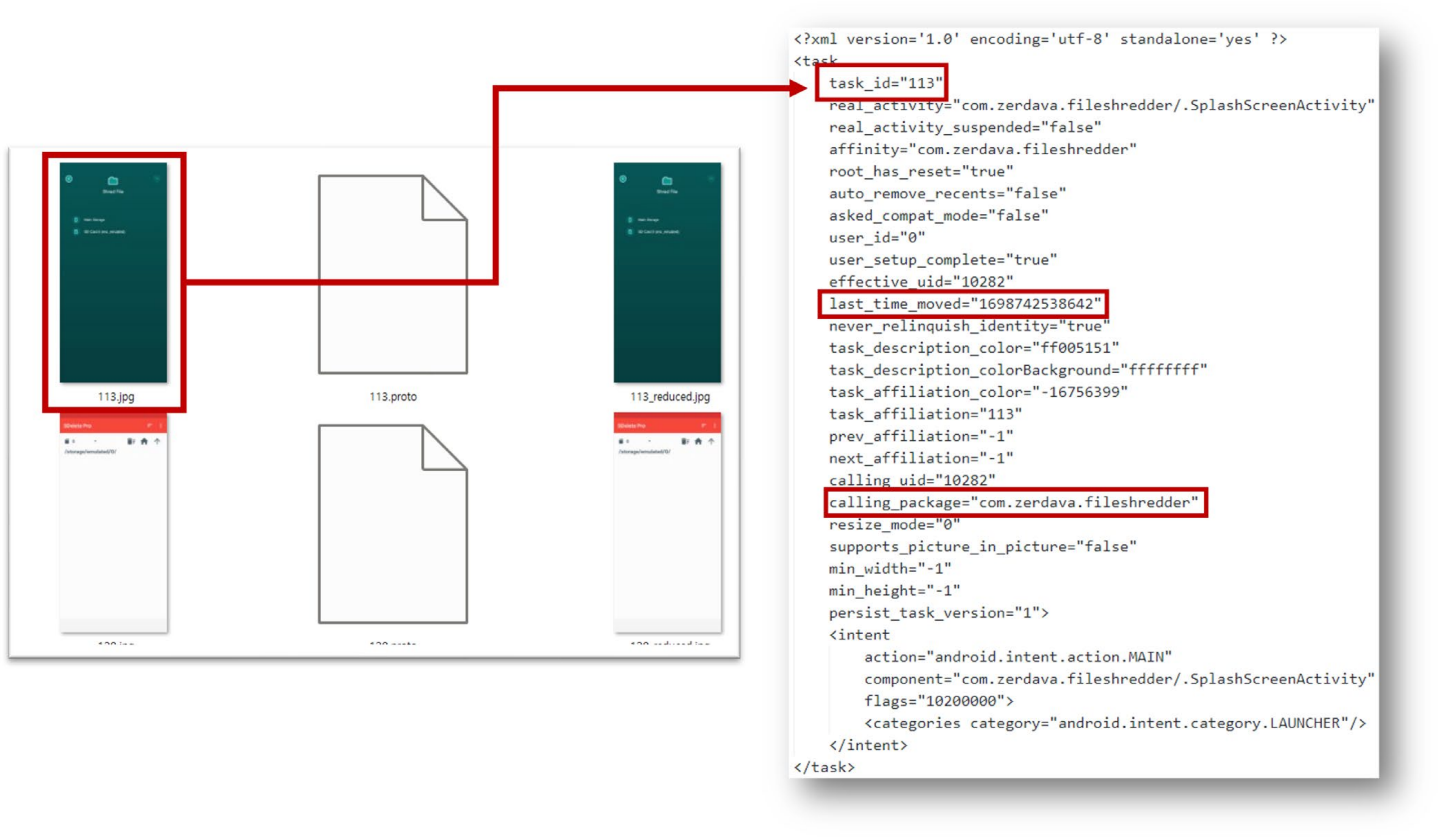
Recent Tasks can provide the names of the recently used packages and their last usage time. And Snapshots gave the last screens of the applications. This figure shows the method of joining two artifacts to gather the last screen of the file‐wiping application.

Figure 10 shows that, when ZERDAVA is running as a background process, we can gather related information through the artifacts left in the Recent Tasks. First, we can identify the package name through the entry “calling package=‘com.zerdava.fileshredder’.” Additionally, by examining “last time_moved=1698742538642,” we can determine that the last execution time was represented as Unix epochs indicating October 31, 2023, at 08:55:38 (GMT + 0).

This entry also includes a “task id” that indicates the task ID of the process in which ZERDAVA is running. Using the identified task ID, we can navigate to the path where Snapshots artifacts are stored and view the last screen displayed by the wiping application. This final screen can provide information that the wiping application performs at that time.

#### 7.2.1 Configuration of file‐wiping applications

SharedPreferences is used on Android OS to store internal configurations in XML format [34]. We found that Data Eraser App and iShredder store settings related to file‐wiping in “/data/data/{package name}/shared_prefs.” By analyzing the key‐value pairs in the XML files (com.cbinnovations.Androideraser.xml for Data Eraser App and com.projectstar.ishredder.Android.standard_preferences.xml for iShredder), we can determine the start and end times of the wiping process, the names of the files being wiped, the wiping algorithm used, and whether the wiping was successful.

**FIGURE 11:**
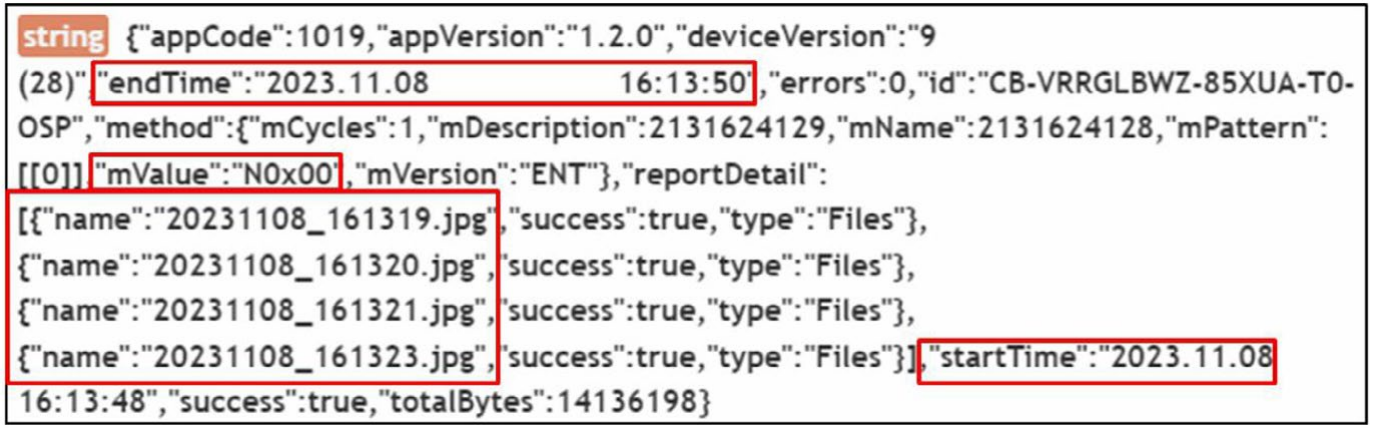
SharedPreferences is used on Android OS to store internal configurations in XML format. We can determine the start and end times of the wiping process, the names of the files being wiped, the wiping algorithm used, and whether the wiping was successful by analyzing this figure.

In Figure 11, the XML data reveal the application version (1.2.0), the Android device version (9), the wiping start time (2023/11/08 16:13:48), and the end time (2023/11/08 16:13:50). It also shows the wiping algorithm used (wiping with 0x00), the number of overwrite cycles (1 cycle), the names of the wiped files (20231108_161319.jpg, 20231108_161320.jpg, 20231108_161321.jpg, 20231108_161323.jpg), and the total bytes successfully overwritten (14,136,198 bytes).

#### 7.2.2 Image cache data in file‐wiping applications

**FIGURE 12:**
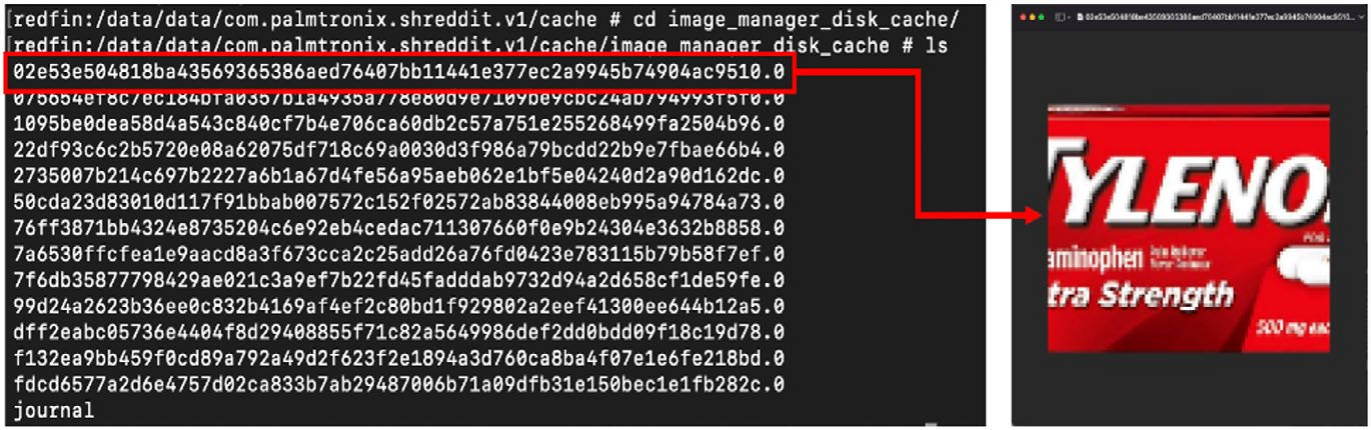
Even if the original image is shredded through file‐wiping, the image cache generated by the file‐wiping application can remain, so this figure shows the process of recovering a wiped image from cached image files.

Shreddit and Secure Wipe Out provide a preview feature that allows users to select which image files to wipe. During the creation of these previews, cache files are also created in the path “/data/data/{package name}/cache/image manager_disk_cache.” Each cache file has a 64‐character name consisting of “0” to “f” and has the extension “0.” To view the content of these cache files, you can change the extension to “jpg,” as shown in Figure 12, which allows to see the preview version of the original image.

This result shows that even if the original image is shredded through file‐wiping, the image cache generated by the file‐wiping application can remain, allowing you to infer the original wiped image.

We also determined the naming principle for these 64‐character filenames. Through reverse engineering, we found that the SHA256 hash function is used, and this hash value can change depending on the model of device used, the original file's name and path, and the original file's content.

Using this information, we propose a new process to identify images suspected of being wiped through the following steps:

1. On the target device, generate a cache for all image files, using the same device model, file‐wiping applications, and filenames.
2. Create a set of these cache files.
3. Compare this set with the set of image caches within the location of cache data in the wiping application.
4. The difference between these sets can help identify images that have been wiped.

Although it is impossible to determine the exact data hashed from the filename due to the irreversible nature of the SHA256 hash function, this process allows the acquisition of information about images suspected of wiping and the inference that wiping has occurred.

Finally, we summarize the observed artifacts and their forensic relevance in Table 6.

**TABLE 6:** Forensic artifacts and their characteristics.

Artifact type	Storage location	Purpose/use in forensics	Example applications
UsageStats	/data/system/0/usagestats/{UNIX TIMESTAMP}	Logs package name, last used time, total run time	All apps
Recent tasks	/data/system_ce/0/recent_tasks/*.xml	Shows recent app activity, package names, and task IDs	All apps
Snapshots	/data/system_ce/0/snapshots/*.jpg	Saves last screen of active apps as JPEG images	All apps
SharedPreferences	/data/data/{package_name}/shared_prefs/*.xml	Stores wiping configuration: algorithm, cycles, filenames	iShredder, Data Eraser App
Image cache	/data/data/{package_name}/cache/image_manager_disk_cache/[0‐f]{64}.0	Retains previews of images selected for wiping	Shreddit, Secure wipe out

## 8 FILE‐WIPING APPLICATION EVALUATION FRAMEWORK

### 8.1 Process of the proposed framework

Based on our comprehensive analysis of wiping artifacts, we have developed an evaluation framework that processes forensic android images to identify file‐wiping tool usage. Although users of file‐wiping tools typically aim to minimize their digital traces, artifacts still remain from OS operations, filesystem activities, and application usage. Based on these residual traces, we can analyze and evaluate wiping tools from an anti‐forensic perspective. The framework operates through three distinct criteria. If an application fails to fully satisfy any of these three criteria, it is evaluated as an incomplete file‐wiping application.

1. Analyze application artifact: Collecting and examining artifacts left by file‐wiping applications to check for data‐wiping logs or caches to find out the execution trace of the wiping application.
2. Tracing the execution of application: Verify compliance with file sanitization mechanisms by hooking wiping functions to confirm compliance with guidelines.
3. Checking residue data in filesystem: Analyze the filesystem to verify data remanence by comparing the data before and after file‐wiping, specifically examining whether any traces of the original file data continue to exist.

However, only when an application fails to meet the “Tracing the Execution of Application” criteria does our framework classify it as an incomplete file‐wiping application, while still acknowledging it as anti‐forensically safe.

### 8.2 Case study Androshredder

For testing our framework, we found another file‐wiping Android application “Androshredder”(MD5:a8f34a489b1d3c035b3a27e8c359de53) in APKpure (https://apkpure.com/). The Androshredder cannot be found in the Google Play Store, and its last update was March 19, 2018. We installed Androshredder on the same devices and under identical experimental conditions as our previous tests and performed file‐wiping. We then evaluated the remaining artifacts and the wiping actions from an anti‐forensic perspective based on our constructed framework.

First, in the “Analyze Application Artifact” criteria, we confirmed that Androshredder did not leave shared_pref.xml or wiping activity caches or image caches in its application internal folder, demonstrating its capability to minimize usage traces. In “Tracing the Execution of Application,” we used Frida's hooking to examine the key wiping functions. While Androshredder was supposed to implement standards like US DoD 5220.22‐M (ECE) which requires single‐value overwriting in first, second, fourth, and fifth passes, we found that it only used random patterns for all wiping standards. This confirmed that Androshredder does not comply with the data sanitization guidelines. Finally, we examine the filesystem residue data before and after wiping, confirming that the file areas were overwritten with random patterns as identified in the previous criteria, making data recovery impossible.

Using our proposed framework from an anti‐forensic perspective, we found that Androshredder left no traceable evidence of wiping activities in the filesystem or application internals, except for OS artifacts related to application execution. However, while implementing data sanitization guidelines should involve using single values for wiping patterns in certain cases, Androshredder ignored this requirement and exclusively used random patterns. Nevertheless, we evaluated that this violation does not compromise the unrecoverability of the wiped data.

## 9 DISCUSSION

### 9.1 Ideal file‐wiping

In this study, we define ideal file‐wiping as a process that not only makes data unrecoverable by overwriting it but also removes any leftover information that could reveal what was deleted or how the wiping was done. This definition is based on an anti‐forensic viewpoint, where the goal is to leave no trace and make investigation difficult. While this may benefit users concerned with privacy or malicious intent, it creates challenges for digital forensic analysts.

We examined six Android file‐wiping applications and saw leftover data such as file names, cached images, and wiping history could still be found after the wiping process. We also found that different wiping patterns leave different clues. Random patterns can look like encrypted or compressed data, making them harder to identify. By contrast, single‐value patterns, particularly uncommon values like 0x01 to 0xFE, are easily identifiable as evidence of wiping. Therefore, these patterns are not effective in hiding wiping activity.

To meet the goal of ideal file‐wiping, it is important to both overwrite the original data and clean up related files such as caches, temporary data, and logs to avoid leaving recoverable traces.

### 9.2 Limitations

However, while the trim commands in filesystems and garbage collection mechanisms in modern operating systems [35] increase the likelihood that deleted data blocks are eventually overwritten they do not guarantee secure erasure—especially in cases where file‐wiping applications, such as Secure Wipe Out, merely simulate shredding but actually perform basic file deletion.

In addition, newer Android versions introduce security enhancements such as Scoped Storage and File‐Based Encryption (FBE). These features restrict access to app‐private directory or raw data, potentially limiting the recoverability of artifacts that were observable in our experiments. As our analysis was conducted on Android devices with root access, further validation is needed to confirm whether similar artifacts persist and can be extracted under stricter, unrooted environments.

Finally, our study focused exclusively on file‐level wiping, although many of the wiping applications also support wiping contacts, messages, free space, and partitions. Another limitation is the small sample size—only six applications—that may not fully reflect the diversity of Android file‐wiping tools. Furthermore, future updates to these applications may change their wiping behavior or artifact generation, potentially affecting the generalizability of our findings.

## 10 CONCLUSIONS

To hinder digital forensic investigations on the Android OS, anti‐forensic techniques such as “file encryption” and “permanent file deletion” can be easily found on the Google Play Store through keyword searches. File‐wiping differs from other anti‐forensic techniques like data hiding or encryption, as it makes recovering the original data extremely difficult even through anti‐anti‐forensic, thus complicating investigations.

Our study aimed to evaluate the effectiveness of file‐wiping applications by validating three research questions and conducting experiments. The results showed that the Secure Wipe Out application, despite claims that it provides a wiping function, only performed file deletions without achieving the intended irrecoverable deletion. Furthermore, we found that many of the wiping algorithms implemented in these applications did not comply with existing guidelines or standards.

In addition, we investigated artifacts generated by file‐wiping activities. By examining UsageStats, we could determine when a specific application was executed and what actions were performed. By using Recent Tasks and Snapshots, we were able to see not only the recent execution times but also the last screen displayed by the application. We also discovered artifacts generated within the file‐wiping applications themselves. In Data Eraser App and iShredder, the application settings were stored in XML format via SharedPreference, allowing us to identify which files were wiped and how. In Shreddit and Secure Wipe Out, the image cache files within the application folders indicated which images had been wiped, as these applications provided a preview feature for selecting images to wipe.

Future research will explore not only the file‐wiping functions, but also partitions and free space wiping, as well as file‐wiping applications on iOS devices. We also aim to collect more wiping applications, conduct experiments across more versions and models, and enhance the generality of our research to develop a robust framework for effectively countering anti‐forensic activities on mobile devices.

## CONFLICT OF INTEREST STATEMENT

The authors declare that they have no known competing financial interests or personal relationships that could have appeared to influence the work reported in this paper.

## Data Availability

The data that support the findings of this study are available from the corresponding author upon reasonable request.
